# Supplementary material for: Mosquitoes on a chip—environmental DNA-based detection of invasive mosquito species using high-throughput real-time PCR
Source: PeerJ. 2024 Sep 30;12:e17782. doi: 10.7717/peerj.17782 (PMC11448751; doi:10.7717/peerj.17782)
Supplement: Supplemental Information 7 [file peerj-12-17782-s007.docx]

| **No.** | **Sampling location** | **Coordinates^1^** | **Sampling date** | **Filter medium/pore size and**  **filtration volume [mL]** | | |
| --- | --- | --- | --- | --- | --- | --- |
|  |  |  |  | **Sterivex®**  **(0.45 µm)** | **Nylon**  **(0.22 µm)** | **PES**  **Smith Root**  **(1.2 µm)** |
| 1 | Cemetery WI Südfriedhof | N°50.060174 E°8.267995 | 30-08-2022 | 200 | 200 | 200 |
| 2 | Cemetery WI Igstadt | N°50.134225 E°8.298470 | 30-08-2022 | 200 | 200 | 200 |
| 3 | Cemetery WI Naurod | N°50.084393 E°8.330092 | 30-08-2022 | 200 | 200 | 200 |
| 4 | Cemetery Ginsheim | N°49.964048 E°8.355783 | 30-08-2022 | 200 | 200 | 200 |
| 5 | Open container, Ketsch | N°49.366570 E°8.533186 | 15-09-2022 | 240 | / | / |
| 6 | Cable duct, Ketsch | N°49.366570 E°8.533186 | 24-10-2022 | 200 | / | / |
| 7 | Watering can, Ketsch | N°49.366570 E°8.533186 | 13-09-2022 | 180 | / | / |
| 8 | Rainwater tank, Bürstadt-Bobstadt | N°49.664232 E°8.445924 | 13-09-2022 | 400 | / | / |
| 9 | Grave vase Bürstadt | N°49.647386 E°8.455347 | 15-09-2022 | 215 | / | / |
| 10 | *Ae. albopictus* laboratory breeding water 1 | N°49.366570 E°8.533186 | 21-10-2022 | 625 | / | / |
| 11 | *Ae. albopictus* laboratory breeding water 2 | N°50.082860 E°8.241343 | 15-09-2022 | 380 | / | / |
| 12 | *Ae. albopictus* laboratory breeding water 3 | N°49.366570 E°8.533186 | 15-09-2022 | 230 | / | / |
| 13 | *Ae. albopictus* laboratory breeding water 4 | N°49.366570 E°8.533186 | 10-10-2022 | 180 | / | / |
| 14 | *Ae. albopictus* laboratory breeding water 5 | N°49.366570 E°8.533186 | 10-10-2022 | 690 | / | / |

^1^Coordinates of sampling sites are given for the center of each site. On sampling sites No. 1-4 divers open water basins were sampled.
