# Supplementary material for: Mosquitoes on a chip—environmental DNA-based detection of invasive mosquito species using high-throughput real-time PCR
Source: PeerJ. 2024 Sep 30;12:e17782. doi: 10.7717/peerj.17782 (PMC11448751; doi:10.7717/peerj.17782)
Supplement: Supplemental Information 8 — Tissue-derived DNA was used for in vitro tests. Positive (+) or failed amplification (−) results are provided with obtained Ct values at 10 ng/μ l measured via qPCR analysis. [file peerj-12-17782-s008.docx]

| Subfamily | Tribe | Genus | Species | AA assay | AJ assay | AK assay |
| --- | --- | --- | --- | --- | --- | --- |
| Culicinae   \| Aedes albopictus \| \| --- \| \| Aedes japonicus \| \| Aedes koreicus \| \| Aedes aegypti \| \| Aedes cantans \| \| Aedes cinerus \| \| Aedes vexans \| \| Anopheles claviger \| \| Anopheles maculipennis \| \| Anopheles plumbeus \| \| Culex pipiens \|   des albopictus | **Aedini** | *Aedes*   \| Aedes albopictus \| \| --- \| \| Aedes japonicus \| \| Aedes koreicus \| \| Aedes aegypti \| \| Aedes cantans \| \| Aedes cinerus \| \| Aedes vexans \| \| Anopheles claviger \| \| Anopheles maculipennis \| \| Anopheles plumbeus \| \| Culex pipiens \|   des albopictus | *albopictus* | + (Ct-value: 16.54) | - | + (Ct-value: 37) |
|  |  | *Aedes* | *japonicus* | - | + (Ct value: 33) |  |
|  |  | *Aedes* | *koreicus* | - | - | + (Ct value: 23.84) |
|  |  | *Aedes* | *aegypti* | - | - | - |
|  |  | *Aedes* | *cantans* | - | - | - |
|  |  | *Aedes* | *cinerus* | - | - | - |
|  |  | *Aedes* | *vexans* | - | - | - |
|  | **Culicini** | *Culex* | *pipiens* | - | - | - |
| Anophelinae |  | *Anopheles* | *claviger* | - | - | - |
|  |  | *Anopheles* | *maculipennis* | - | - | - |
|  |  | *Anopheles* | *plumbeus* | - | - | - |
|  | Negative control | |  | - | - | - |
|  | Human DNA | |  | - | - | - |
