# Supplementary material for: Mosquitoes on a chip—environmental DNA-based detection of invasive mosquito species using high-throughput real-time PCR
Source: PeerJ. 2024 Sep 30;12:e17782. doi: 10.7717/peerj.17782 (PMC11448751; doi:10.7717/peerj.17782)
Supplement: Supplemental Information 9 [file peerj-12-17782-s009.docx]

| Species | formula | slope | efficiency | R^2^ | LOQ | LOD |
| --- | --- | --- | --- | --- | --- | --- |
| *Ae. albopictus* | y = -1.652ln(x) + 31.898 | -3.59 | 90% | 0.99 | 36.41 | 37.47 |
| *Ae. japonicus* | y = -1.457ln(x) + 35.05 | -3.35 | 99% | 0.99 | 34.96 | 38.95 |
| *Ae. koreicus* | y = -1.515ln(x) + 32.471 | -3.49 | 94% | 0.99 | 32.20 | 36.58 |
