# Supplementary material for: Mosquitoes on a chip—environmental DNA-based detection of invasive mosquito species using high-throughput real-time PCR
Source: PeerJ. 2024 Sep 30;12:e17782. doi: 10.7717/peerj.17782 (PMC11448751; doi:10.7717/peerj.17782)
Supplement: Supplemental Information 10 — Letters A or B indicate the biological replicates from which the sequences were obtained. [file peerj-12-17782-s010.docx]

| **Species** | **Sample name** | **Sequence 5’-3’** |
| --- | --- | --- |
| *Ae. albopictus* | Open container, Ketsch | GTCAGCAGGGCCGAACCCGCGCAGGGCACATACGTCCGCTTTGGTTTGACATAGCTAAGTGGCGGGTC |
|  | *Ae. albopictus* laboratory breeding water 2 | GTCAGCAGGGCCGAACCCGCGCAGGGCACATACGTCCGCTTTGGTTTGACATAGCTAAGTGGCGGGTC |
|  | Ae_albopictus_tissue | GTCAGCAGGGCCGAACCCGCGCAGGGCACATACGTCCGCTTTGGTTTGACATAGCTAAGTGGCGGGTC |
| *Ae. japonicus* | WI Igstadt_NylonA | GCTCCAGATATAGCTTTCCCTCGAATAAATAATATAAGTTTTTGAATATTACCCCCTTCTTTAACCTTACTACTTTCAAGTAGAATGGTAGAAAATGGATCTGGAACTGGATGAACTGTTTATCC |
|  | Open container, Ketsch | GCTCCAGATATAGCTTTCCCTCGAATAAATAATATAAGTTTTTGAATATTACCCCCTTCTTTAACCTTACTACTTTCAAGTAGAATGGTAGAAAATGGATCTGGAACTGGATGAACTGTTTATCC |
|  | Ae_japonicus_tissue | GCTCCAGATATAGCTTTCCCTCGAATAAATAATATAAGTTTTTGAATATTACCCCCTTCTTTAACCTTACTACTTTCAAGTAGAATGGTAGAAAATGGATCTGGAACTGGATGAACTGTTTATCC |
| *Ae. koreicus* | WI Südfriedhof_NylonA | CCCAGATATAGCCTTCCCCCGAATAAATAATATAAGTTTTTGAATACTACCTCCCTCATTAACTCTACTACTTTCAAGAAGTATAGTAGAAAATGGGTCTGGGACAGGATGAACTGTTTATCC |
|  | Ae_koreicus_tissue | CCCAGATATAGCCTTCCCCCGAATAAATAATATAAGTTTTTGAATACTACCTCCCTCATTAACTCTACTACTTTCAAGAAGTATAGTAGAAAATGGGTCTGGGACAGGATGAACTGTTTATCC |
